# “Heads Up Girls!” a training intervention to improve scanning behavior in youth female football

**DOI:** 10.3389/fspor.2025.1602099

**Published:** 2025-06-27

**Authors:** Mirjam Hintermann, Michael Romann, Dennis-Peter Born, Wolfgang Taube, Jörg Fuchslocher

**Affiliations:** ^1^ Department of Elite Sport, Swiss Federal Institute of Sport Magglingen SFISM, Magglingen, Switzerland; ^2^ Department of Neurosciences and Movement Sciences, University of Fribourg, Fribourg, Switzerland; ^3^ Swiss Development Hub for Strength and Conditioning in Swimming, Swiss Aquatics—National Swimming Federation, Worblaufen, Switzerland

**Keywords:** female athletes, visual exploration, training intervention, small-sided games, football

## Abstract

**Background:**

In football, visual exploration and scanning are crucial to make players aware of their teammates’ and their opponents’ positions, thus improving their decision-making. However, specific recommendations on systematic training methods to improve scanning behavior are limited. Therefore, this study investigated the effects of a five-week scanning-focused training intervention on scanning behavior in U19 female football players.

**Methods:**

A total of 36 female football players (mean ± SD age: 16.7 ± 1.2 years) from two elite and two grassroots teams were assigned to a control and intervention group. The intervention group completed one to two scanning-focused training sessions per week, while the control group followed their regular training routine. Scanning behavior was assessed using video analysis of 4v4 small-sided games in pre-, post-, and retention tests. A scan was defined as an active head movement, during which the player's head was directed away from the ball.

**Results:**

The intervention group significantly increased mean number of scans per game situation from pre- to post-test (*p* = 0.002), with no significant improvements in the control group (*p* = 0.088). However, this improvement was only observed in elite (*p* = 0.001), but not in grassroots players. After scanning, elite players performed more successful actions than grassroots players (*p* = 0.011), with no significant effect throughout the intervention period (*p* = 0.074). The retention test three weeks later (*n* = 31) showed that the increased scanning behavior of the post-test was maintained.

**Conclusion:**

In conclusion, a five-week scanning-focused training intervention improves scanning behavior in young female football players, particularly at elite level. These findings highlight the value of integrating perceptual-cognitive training into football coaching.

## 1 Introduction

Football is a complex ball game with a constantly changing environment, i.e., continuously moving teammates, opponents, and ball. Visual exploration may improve players awareness of their surroundings and promote adequate decision-making (1). According to Jordet et al. (2), scanning is an essential, information-gathering process during which players must temporarily direct their heads (and eyes) away from the ball to scan for teammates, opponents, and open space. This scanning behavior has been identified as a key component of expert performance, with elite players demonstrating a higher scanning frequency before ball reception than less-skilled players (2–6). Previous research in real game settings examined scanning across age groups (3), skill levels (6), contextual factors (2, 3, 7), and timing relative to ball reception (1, 8, 9), with a focus on the relationship between scanning and performance outcomes. Evidence suggests that scanning prior to ball reception increases the likelihood of a successful subsequent action (2, 5, 6, 10, 11). However, almost all existing research on scanning behavior has been conducted in men's football. Beyond well-established physiological and physical differences between the sexes that influence game dynamics (12), recent studies have revealed sex-specific patterns in perceptual-cognitive abilities and training responses. For example, Legault and Faubert (13) found that male athletes had superior baseline perceptual abilities compared to female athletes, both sexes showed significant improvements after short-term perceptual-cognitive training. Yongtawee and Woo (14) demonstrated that training experience affects cognitive development differently across sex, with male athletes showing greater benefits in interceptive sports. Therefore, given their unique training histories and developmental trajectories, it is important to study female players as a distinct group. Moreover, given that the number of registered female football players in Europe has doubled since 2019 (15), research on female players has gained increasing attention (16), although it is still minimal compared to that in male footballers. The limited research on scanning behavior in female central midfield players participating in the UEFA Women's 2022 European Championships suggests that their scanning frequency was lower than that of male players (17). More specific analyses demonstrated that the competitive level affects scanning, with U19 elite female players scanning more frequently than their grassroots counterparts in 4v4 small-sided games (SSGs) (6).

Although scanning is widely recognized as a critical perceptual skill in elite football, research on specific recommendations of how to systematically develop and improve scanning in football is limited. Pulling et al. (18) found that the most commonly used method to integrate scanning in training sessions is direct instructions, i.e., as verbal cues (“head up”) or questioning the players about their scanning behavior. Additionally, task constraints, such as modified pitch dimensions, varying number of players, or additional targets, which include opponent pressure and real game scenarios are common strategies to increase players’ scanning behavior (19). Small-sided and conditioned games are well-documented training formats that improve technical and tactical skills (20). McGuckian et al. (21) demonstrated that reducing available space per player in 3v3 SSGs led to increased scanning compared to full-size (11v11) pitches, both with and without possession of the ball. Elderidge et al. (22) found that players scanned more frequently in a passing exercise without opponents (using marked bibs to denote teammates and opponents) compared to in-game SSG. Furthermore, some studies have explored visual occlusion training, using spatial occlusion goggles to improve response time and accuracy (23, 24). However, such artificial constraints may have limited transferability to real-game scenarios, where representative learning design is crucial for ecological validity (25). As such, training interventions should reflect real-game conditions to allow players to naturally perceive affordances, i.e., possibilities for action that emerge dynamically within the game (26). To achieve ecological validity, football training should expose players to dynamic, game-representative scenarios that challenge both their technical execution and tactical awareness, thereby enhancing the transfer of adaptations to competitive play (27). One widely used method of replicating match play in training is the use of SSGs, which may serve as an effective setting for developing perceptual skills, such as scanning.

Due to the aforementioned limited research, coaches continue to rely on trial-and-error approaches and lack structured, empirically supported training methods. Additionally, most research on scanning has been cross-sectional, with little emphasis on systematic training interventions. This study adds to the literature by providing empirical evidence on the trainability of scanning behavior and its performance implications in female football. Therefore, the aim of the study was to investigate the effects of a five-week scanning-focused training intervention on scanning behavior in U19 female football players. Additionally, the study assessed whether potential training effects were maintained in a retention test three weeks after the intervention. We hypothesized that scanning behavior would improve after the five-week scanning-focused training intervention and that a larger number of scans after the intervention would increase the success rate of subsequent actions.

## 2 Methods

### 2.1 Participants

Sixty-one female football players from two Swiss U19 women's national league teams and two U19 grassroots teams volunteered to participate in this study. A total of 36 players (mean ± SD age: 16.7 ± 1.2 years; Team 1: *n* = 9, Team 2: *n* = 10, Team 3: *n* = 12, Team 4: *n* = 5) met the inclusion criteria: (1) participation in both pre- and post-test, (2) participation in at least five intervention training sessions, (3) outfield playing position (goalkeepers were excluded). On average, elite players had 10.1 ± 1.9 years and grassroots players had 5.4 ± 3.0 years of football experience. Written informed consent was obtained from all participants or their legal guardians in the case of the underage participants. The ethical aspects of the study were approved by the institutional review board of the Swiss Federal Institute of Sport Magglingen SFISM (196_LSP_04_2023) and the study was conducted in accordance with the latest version of the Declaration of Helsinki.

### 2.2 Training intervention

Training content was designed in collaboration with the participating teams’ coaches to foster scanning development within a representative learning design. Thus, four game formats with variations and four exercises were developed to expose players to a wide range of game situations and sudden changes, such as shifts in game direction or moving visual cues that need to be identified during play. These conditions required players to make quick decisions and continuously reassess the game environment. Table 1 provides an overview of the game formats and exercises from the playbook used during the scanning-focused training designed to enhance scanning behavior in the intervention group. Details are provided on the team setups, objectives of the exercises and game formats, and key training elements. The detailed instructions are provided in the supplementary information (Supplementary File 1).

**Table 1:** Overview of game formats and exercises in the scanning-focused training intervention.

	Game 1	Game 2	Game 3	Game 4	Exercise 1	Exercise 2	Exercise 3	Exercise 4
Team	4v4 + neutral player	4v4/5v5	4v4 + goalkeepers	4v4 + goalkeepers	Circle with 5 outside and 1 central player	Groups of 3 players	Groups of 3–4 players	3 teams of 4 players
Goal	–	6 small goals	2 regular goals	2 regular goals	–	–	–	–
Objective	- Keep ball possession - Recognition of visual cues	- Quick transitions and awareness of open goals	- Free directional play after crossing halfway line	- Controlled buildup from the goalkeeper - Play behind the player	- Quick decision-making and passing in movement	- First touch control and passing accuracy under constraints	- Scanning and adjusting position - Recognition of visual cues	- Decision-making based on teammate communication

Before implementing the intervention, exercises and game formats were pre-tested with an independent team to refine instructions and optimize the new playing formats. Based on these pre-tests, coaches of the participating teams received printed instructions detailing all exercises, game formats, and corresponding instructions. Before the start of the study, coaches were introduced to the game formats through pre-tested video footage and personal on-field instructions allowing the coaches to clarify any potential questions. During the intervention period, coaches lead the scanning-focused training sessions in order to maintain normal training conditions and natural player behavior. For each scanning-focused training session, the coaches could choose the exercises and game formats (including at least one game format) to match the overall focus of the training session. For intervention fidelity, coaches documented each training session immediately afterward. Reports included details on selected exercises, game formats, modifications, duration, remarks, and participating players. These reports were descriptively reviewed by the study leader to ensure consistent implementation of the intervention across teams and to confirm appropriate variation in selected exercises and formats from the playbook. No substantial differences between the teams or outliers were identified. Furthermore, each team was observed twice from the sideline by the study leader during intervention training sessions, without any interference. Post-training discussions with coaches were conducted without the players’ presence to ensure an open exchange of feedback and observations. Slight modifications (mainly refined instructions) to exercises and game formats were conducted based on coaches’ feedback throughout the intervention period to further refine the study approach. These adaptations were primarily designed to help coaches manage variations in player numbers or infrastructure and ensure consistent training conditions throughout the intervention.

### 2.3 Procedures

As the teams were located up to 90 km apart from each other, players were assigned to either the control or intervention group based on their team affiliation, following a quasi-experimental rather than completely randomized design. Both the control as well as the intervention group consisted of one grassroots and one elite team. The intervention was conducted over five weeks, including one to two scanning-focused training sessions per week. Only one scanning-focused training session was conducted in weeks with two official matches. This was the case for the elite teams in two out of the five-week intervention period. Each scanning-focused training session included a 30-minute block of scanning-focused exercises and game formats, which was conducted immediately following the warm-up. During that 30-minute block, the control group followed their regular training routine, emphasizing ball control and first touch within their game formats and exercises. However, they did not engage in scanning-specific game formats and exercises (Supplementary File 2).

### 2.4 Test sessions

Test sessions were conducted one week before (pre-test), one week after (post-test), and three weeks after (retention test) the five-week intervention period. Coaches assigned outfield players from their team to four balanced sub-teams, ensuring equal distribution of physical, technical, and tactical abilities. Players remained in the same sub-teams throughout all test sessions. After a standardized 15-minute warm-up, the test session included two sets of 4 × 4-minute SSG, with a 2-minute break between games and a 5-minute break between the sets. The games were played on two 40 × 30 m pitches, following modified 11v11 rules: no offside rule and goalkeepers were allowed to restart play after goals and out of play situations (6). Throughout the session, multiple balls were available for the out-of-play situations and were made available immediately by coaches positioned around the pitch to maintain continuous gameplay. A 1:2:1 playing system was implemented to ensure that players rotated through all four positions within each set and to minimize position-specific effects on performance. All 4v4 games were manually recorded using two 4 K digital cameras (HDR-CX700VE, Sony, Minato, Tokyo, Japan). The cameras were mounted on tripods and positioned 2 m from the long side and 7 m from the short side of each pitch. No zoom was applied during filming to maintain a consistent field of view.

### 2.5 Variables

#### 2.5.1 Scanning

A scan was defined as an active head movement during which the player temporarily turns their face away from the ball to assess the positions of teammates, opponents, or available space for subsequent playing actions (2). The video footage was used to analyze scanning behavior for all game situations during which players received a pass from a teammate (3). Those game situations were defined as follows: from 5 s before the first ball contact until the ball was released for a subsequent action (pass, dribbling, or shot) (6).

#### 2.5.2 Skill level

Skill level was defined based on the league in which the players trained and competed. Elite players participated in the U19 Swiss National Women's League, while grassroots players competed in the U19 female grassroots league.

#### 2.5.3 Performance of subsequent actions

Scanning behavior was assessed based on the effectiveness of each subsequent performance. The subsequent action was considered successful, if the intentionally played ball reached a teammate or ball-possession was maintained within the team. Subsequent actions were classified as “unsuccessful”, if the intentionally played ball left the pitch boundaries or was intercepted by the opposing team, i.e., loss of ball-possession. The performance of subsequent actions was expressed as the percentage of positive outcomes.

### 2.6 Data analysis and statistics

Filming and tagging procedures followed the methodology outlined by Hintermann et al. (6). All video footage was analyzed using the same tagging panel as in the previous study and assessed by the same two expert raters. Interrater reliability was assessed using intraclass correlation coefficients (ICCs), calculated via mixed-effects models under the assumption of absolute agreement with a single rater. Estimates and 95% confidence intervals (95% CI) were computed using the psych-package in R (28). A randomly selected subset of 108 game situations (4% of total) from four games involving four players was independently analyzed by both raters. The resulting ICC was 0.89 [95% CI (.86,.92)], indicating good to excellent interrater reliability (29). To assess intrarater reliability, each rater independently re-analyzed four games of two randomly selected players (4% of total). The ICCs demonstrated good to excellent intrarater reliability for both raters [rater 1: ICC = 0.86, 95%CI [.79,.90]; rater 2: ICC = .93, 95% CI [.81,.99], respectively] (29).

To address the main research question and account for dropouts, scanning behavior was compared between the intervention and control groups, which pooled elite and grassroots players. Skill level and the percentage of positive subsequent actions were then analyzed in the subgroups. Thirty-one out of 36 players (14 in the intervention and 17 in the control group) participated in the retention test two weeks after the post-test and were analyzed as another subgroup.

All data are presented as mean ± SD and the alpha level set at *p* < 0.05. The assumption of normality was checked through visual inspection, evaluating the predicted and standardized residuals. Normal distribution was confirmed by a random distribution around zero in the scatter plot, a diagonal straight line in the QQ-plot, and a Gaussian distribution in the histogram (30). A two-way repeated measures analysis of variance (ANOVA) with Bonferroni-adjusted *post-hoc* comparisons was calculated to determine potential differences and interaction effects in the mean number of scans per game situation between groups (control vs. intervention) across two or three time points (pre- vs. post- vs. retention test) and two skill levels (elite vs. grassroots). Effect sizes were interpreted using partial eta squared (*η*^2^), with thresholds of 0.01, 0.06, and 0.14 indicated a small, medium, and large effect (31).

To assess the performance of subsequent actions, only game situations with scanning behavior before ball reception were analyzed. Mean percentages of positive outcomes were compared using a two-way ANOVA, with skill level (elite vs. grassroots) and time (pre-test vs. post-test) as independent variables. Additionally, a Mann–Whitney U test was used to compare the mean percentage of positive outcomes between game situations with and without scanning before first ball contact.

## 3 Results

In total, 2,458 game situations were included in the analyses. On average, players in the intervention group had 4.67 game situations per game in the pre-test and 4.91 in the post-test. Players in the control group showed a similar activity level, with 4.65 game situations per game in the pre-test and 4.91 in the post-test.

### 3.1 Scanning behavior

The results of the training intervention are presented in Table 2. In line with our first hypothesis, the intervention group significantly increased their mean number of scans per game situation from the pre- to the post-test (*p* = 0.002). Additionally, during the post-test mean number of scans were significantly higher in the intervention than the control group (*p* = 0.002). During the intervention period the control group also exhibited a slight increase in mean number of scans per game situation, which however, did not reaching statistical significance (*p* = 0.088). The mean changes from pre- to post- (delta) in the number of scans per game situation were 0.55 ± 0.37 (95% CI: 0.37–0.74) in the intervention group and 0.31 ± 0.35 (95% CI: 0.16–0.46) in the control group.

**Table 2:** Mean number of scans per game situation before (pre-) and after (post-) the five-week training intervention.

Mean number of scans per game situation	Training condition		ANOVA			
	Intervention (*n* = 15)	Control (*n* = 21)	*F*-value		*P*-value	Partial eta^2^
Pre-	0.88 ± 0.36 (0.70–1.07)	0.61 ± 0.27 (0.49–0.72)	a)	*F*_(1,68)_ = 19	*p* < 0.001	0.21 (large)
Post-	1.44 ± 0.52 (1.17–1.70)*^,+^	0.92 ± 0.43 (0.73–1.10)	b)	*F*_(1,68)_ = 17	*p* < 0.001	0.20 (large)
			c)	*F*_(1,68)_ = 2	*p* = 0.203	

Data of elite and grassroots players were pooled for this analysis. Significant differences were identified by a 2-way repeated measures analysis of variance (ANOVA): training condition (control vs. intervention)×time (pre- vs. post-). a) Main effect: time (pre- vs. post-); b) Main effect: training condition (control vs. intervention); c) interaction effect: time × training condition. The delta represents the mean of the individual mean changes per player. Data are presented as mean ± SD (95% confidence interval).

^*^ Significant difference compared to control group.

^+^ Significant difference compared to pre-test.

**Figure 1:**
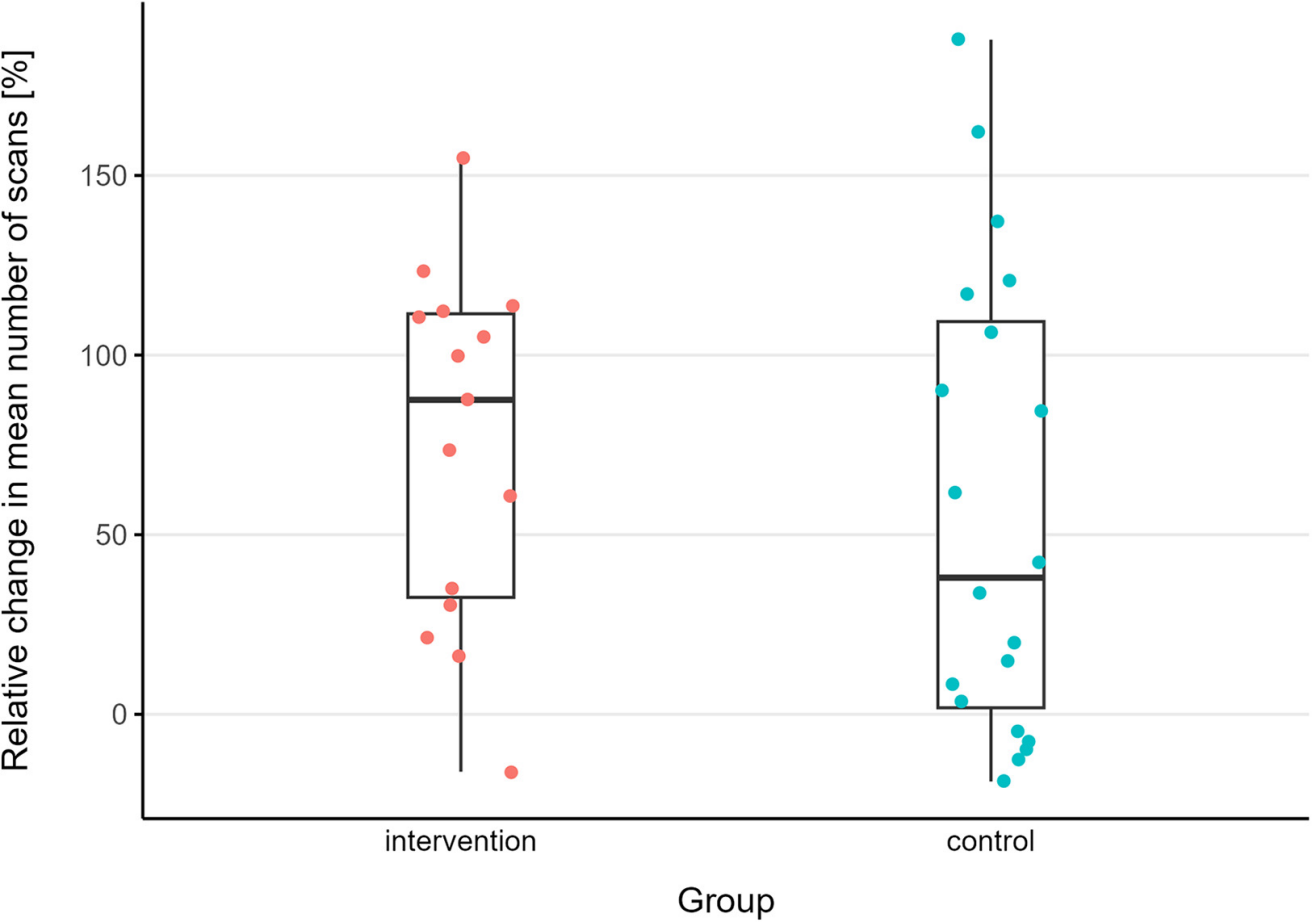
Relative change in mean number of scans per game situation from pre- to post-test for intervention and control group. Boxplot with median ± inter-quartile range and whiskers.

Figure 1 provides an overview of the relative change in the mean number of scans per game situation from pre- to post-test for intervention and control groups.

### 3.2 Percentage of positive subsequent actions

The two-way ANOVA showed that elite players had a significantly higher percentage of positive subsequent actions compared to grassroots players (elite: 75.4%; grassroots: 65.1%; *F*_1,68_ = 7, *p* = 0.011, *η*^2^ = 0.09). There was a trend for time that indicated a potential decrease in percentage of positive outcomes from pre- to post-test, though this effect was not statistically significant (pre-test: 74.3%; post-test: 67.1%; *F*_1,68_ = 3, *p* = 0.074, *η*^2^ = 0.05). This result does not support our second hypothesis, which predicted that an increase in the number of scans would be associated with a higher success rate. However, a comparison of game situations with and without scanning behavior showed a significantly higher percentage of positive outcomes when players scanned before their first ball contact (with scanning: 70.6%; without scanning: 62.5%; *z* = −3.45, *p* = 0.001).

### 3.3 Skill level differences

Differences between elite and grassroots players are presented in Table 3. The table shows mean number of scans per game situation before and after the training intervention for both skill levels, across the intervention and control groups. The two-way ANOVA revealed significant main effects of time (pre- vs. post-; *p* < 0.001) and condition (control vs. intervention; *p* < 0.001) in the elite group. In the grassroots group, a significant main effect of time (*p* = 0.007) but not condition (*p* = 0.367) was found. In elite players, the mean changes (delta) in the number of scans per game situation from pre- to post- were 0.66 ± 0.41 (95% CI: 0.41–0.91) in the intervention group, compared to 0.39 ± 0.43 (95% CI: 0.11–0.67) in the control group. In contrast, in grassroots players, the corresponding mean changes were 0.35 ± 0.12 (95% CI: 0.24–0.45) in the intervention group and 0.25 ± 0.29 (95% CI: 0.09–0.41) in the control group.

**Table 3:** Mean number of scans before (pre-) and after (post-) the five-week training intervention in elite and grassroots players.

Mean number of scans per game situation	Training condition		ANOVA			
	Intervention	Control	*F*-value		*P*-value	Partial eta^2^
Elite group						
Pre-	1.06 ± 0.26 (0.90–1.22)	0.78 ± 0.16 (0.68–0.88)	a)	*F*_(1,34)_ = 25	*p* < 0.001	0.42 (large)
Post-	1.72 ± 0.39 (1.48–1.96)*^,+^	1.17 ± 0.43 (0.89–1.45)	b)	*F*_(1,34)_ = 15	*p* < 0.001	0.31 (large)
			c)	*F*_(1,34)_ = 2	*p* = 0.218	
Grassroots group						
Pre-	0.52 ± 0.24 (0.31–0.74)	0.48 ± 0.26 (0.33–0.63)	a)	*F*_(1,30)_ = 8	*p* = 0.007	0.22 (large)
Post-	0.87 ± 0.12 (0.76–0.98)	0.72 ± 0.34 (0.53–0.91)	b)	*F*_(1,30)_ = 1	*p* = 0.367	
			c)	*F*_(1,30)_ = 0	*p* = 0.638	

Significant differences were identified by a 2-way repeated measures analysis of variance (ANOVA): training condition (control vs. intervention) × time (pre vs. post). a) Main effect: time (pre vs. post); b) Main effect: training condition (control vs. intervention); c) Interaction effect: time × training condition. The delta represents the mean of the individual mean changes per player. Data are presented as mean ± SD (95% confidence interval).

* Significant difference compared to control group.

^+^ Significant difference compared to pre-test.

Among elite players, those in the intervention group performed significantly more scans in the post-test compared to the control group (*p* = 0.005). Additionally, elite players in the intervention group exhibited a significant increase in scanning behavior from pre- to post-test (*p* = 0.001). In contrast, no significant *post-hoc* effects were observed among grassroots players.

**Figure 2:**
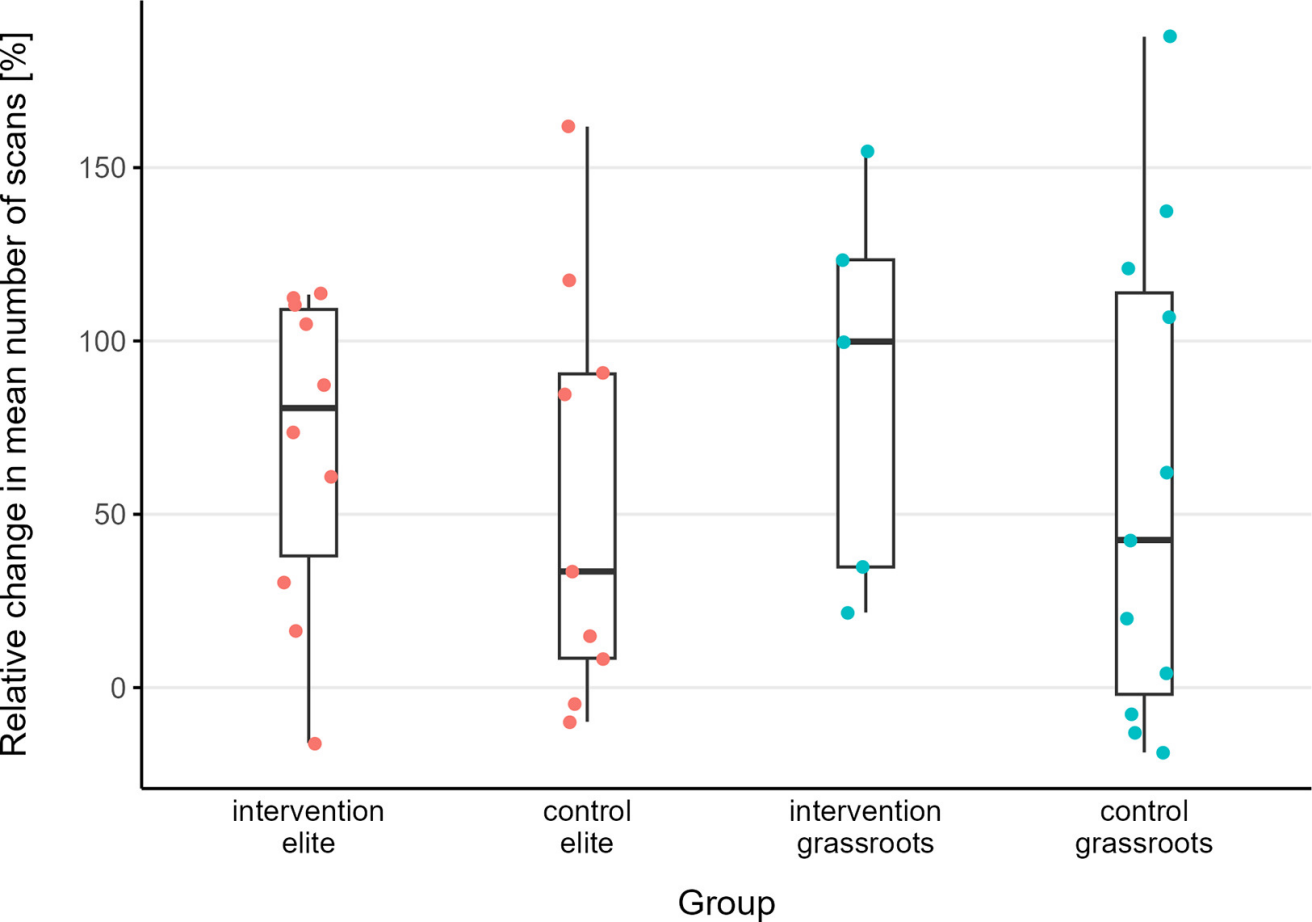
Relative changes in the mean number of scans from pre- to post-test, comparing the control and intervention groups across performance levels. Boxplot with median ± inter-quartile range and whiskers.

An overview of the relative changes in the mean number of scans per game situation from pre- to post-test in both the intervention and control groups, across both performance levels, is presented in Figure 2.

### 3.4 Retention test

The 2-way repeated measures ANOVA (*n* = 31) reported statistically significant main effects of time (pre- vs. post- vs. retention test; *F*_2,87_ = 12, *p* < 0.001, *η*^2^ = 0.21) and condition (control vs. intervention; *F*_2,87_ = 23, *p* < 0.001, *η*^2^ = 0.21). The *post-hoc* test indicated an increase in the mean number of scans in the intervention group from pre- to post-test [pre: 0.86 ± 0.36, 95% CI [0.67; 1.05]; post: 1.47 ± 0.53, 95% CI [1.19; 1.74]; *p* = 0.004] and from pre- to retention test [retention: 1.47 ± 0.55, 95% CI (1.18; 1.75); *p* = 0.004]. As previously shown in Table 2, no significant differences were observed in the control group between pre- and post-measurements. Furthermore, no significant changes in number of scans were detected between the post- and retention test in the intervention or control group. However, there were significant differences (post: *p* = 0.018; retention: *p* = 0.015) in post- and retention test results between the two groups.

**Figure 3:**
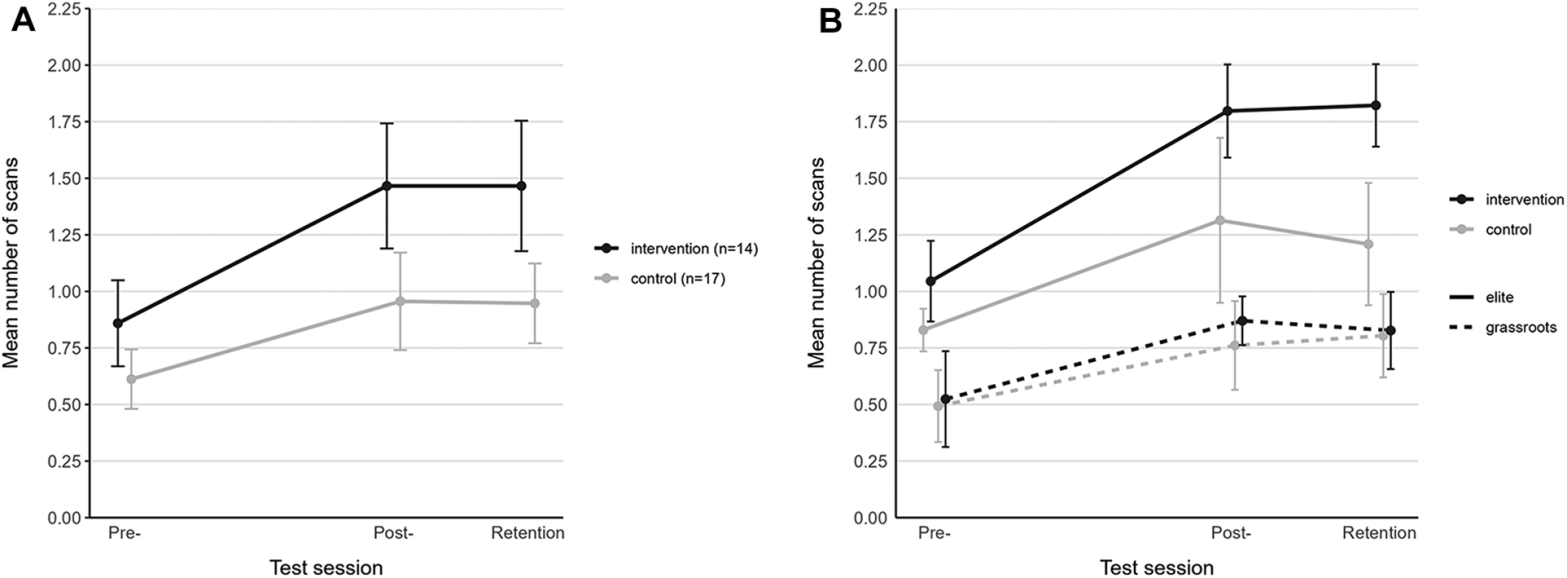
Changes in mean number of scans from pre- to post- and retention test in control and intervention groups **(A)** and across performance levels **(B)**.

The progression in mean number of scans per game situation from pre- to post- and retention test is illustrated in Figure 3, with comparisons across intervention and control groups (Figure 3A) and performance levels (Figure 3B).

## 4 Discussion

This is the first study to investigate the effects of a scanning-focused training intervention on scanning behavior in youth female football players. The main finding is that mean number of scans per game situation significantly increased after five weeks of training in the intervention but not the control group. This improvement was maintained in the retention test. However, scanning behavior only improved in the elite but not the grassroots players. After scanning, the percentage of positive subsequent actions was higher in elite compared to grassroots players and in game situations where players scanned, rather than when they did not.

In line with our first hypothesis, the number of scans per game situation increased following the five-week intervention focused on scanning. However, this increase from pre- to post-test was primarily driven by the elite group, as grassroots players did not exhibit any statistically significant improvements. The larger effect in the elite players may be attributed to their generally more superior motor skills, which they developed through a larger training volume across their career. The resulting technical expertise of elite players may facilitate the ability to turn their head away from the ball to simultaneously perceive relevant environmental information for their subsequent actions (32). This suggests that elite players are more likely to engage brain regions essential for task execution in novel and unexpected situations, thereby demonstrating a greater capacity for scanning than grassroots players (33). Neurological adaptations in the cerebral cortex could play a key role in this process, as extensive motor training has been linked to changes in cortical activation (34, 35). The greater neural efficiency in more skilled players (36) may contribute to an enhanced ability to process and regulate motor information and to acquire new skills more rapidly (37). From a practical perspective, grassroots players may initially benefit from motor skill training to develop and stabilize fundamental football-specific abilities before focusing on their scanning behavior. Further research should investigate how individual skill level affects the effectiveness of scanning-focused training.

From a practical standpoint, the increase of 0.55 in the mean number of scans per game situation observed in the intervention group reflects a meaningful behavioral change. In our 4v4 test format, players typically experience approximately 38 ball receptions per test session. This translates to an increase from about 33 scans in the pre-test to 55 in the post-test, equating to roughly 20 additional scans per player. Coaches involved in the study also reported that players became more aware of their surroundings and made decisions more quickly. These qualitative observations support the practical importance of the increased mean number of scans, demonstrating its relevance beyond statistical outcomes.

Interestingly, the control group also exhibited a trend towards improved scanning behavior from pre- to post-test. This may be partly due to natural skill development in young players, as they continue to refine their perceptual and motor abilities over time. Additionally, tactical and technical exercises and games performed as part of their regular training routine may have had a positive influence on scanning behavior, due to incidental learning mechanisms. Moreover, discussions about scanning and participation in the study itself may have raised awareness, contributing to their improved scanning behavior. Although, the hypothesis should be confirmed with a larger sample size, pointing out the importance of scanning may be a time-efficient alternative to extensive and structured training interventions.

The benefits of the scanning-focused training were preserved for at least three weeks longer as still evident during the retention test. Those long-term effects and maintenance of the improvements show that players are able to internalize and consolidate scanning behaviors (38). These findings align with skill acquisition theories that emphasize the importance of representative learning designs in promoting perceptual-cognitive adaptations that are maintained beyond immediate practice (25). However, effects of the scanning intervention beyond the three-week retention period have yet to be determined.

In our second hypothesis, we assumed that an increased number of scans following the intervention would be associated with a higher success rate of subsequent actions. The present study showed a higher percentage of successful subsequent actions in elite compared to grassroots players, which aligns with previous findings (2). However, despite the improved scanning behavior, which is correlated to success and performance outcomes, there was no change in the percentage of positive outcomes from the pre- to post-test, thus not supporting our hypothesis. In fact, there was even a trend towards a decline in the success rate of the subsequent actions in the post-test. As the primary aim of the study was to improve scanning behavior, players may have increased the number of scans even in very challenging game situations, such as under higher opponent pressure. Therefore, the players may have performed more than the optimal number of scans for such high-pressure game situations. Under such conditions, increased scanning may lead to cognitive overload, where the processing of excessive visual information interferes with motor execution. According to the constrained action hypothesis, excessive conscious control over movements, such as through over-scanning, can disrupt automatic processes and reduce performance (39). In our study, the observed trend of decreased success rates despite increased scanning may reflect this phenomenon. It is also important to note that successful actions in football depend on multiple interacting factors, which makes it difficult to directly decipher the effect of scanning behavior on performance. Still, future research should examine the effect of increased scanning in relation to contextual factors, such as opponent pressure or playing position (2, 3, 7).

### 4.1 Limitations

While the findings of the present study provide valuable insights into the development of scanning behavior in U19 female football players, several limitations should be acknowledged. Firstly, teams were not completely randomly assigned to intervention and control groups. Instead, group allocation was based on team affiliation and regional proximity, which may have introduced selection bias. Secondly, from the original sample size of 61 players, only 36 completed enough training and test sessions to be included in the final data set, which reduces statistical power and generalizability despite significant differences. Thirdly, the study was conducted with U19 female players and future studies have to verify the findings in other age groups and skill levels. Lastly, the retention test was conducted three weeks after the intervention. To investigate long-term sustainability of the observed improvements longer follow-up periods are required.

### 4.2 Practical implications

The results of this study provide helpful guidance for coaches aiming to promote scanning behavior in young female football players. Since significant improvements were only demonstrated by elite players, the study emphasizes the necessity to actively foster and develop scanning behavior in high-performance settings where perceptual-cognitive skills already play a central role (18). Rather than treating scanning as an isolated skill, coaches should consider it an integral component of overall player development. To support this, training environments should follow the principles of representative learning design. For example, SSGs ensure that the practice task simulates the perceptual and decision-making demands of actual match play (25). Due to their adaptability, SSGs are particularly well-suited to manipulate environmental, individual, or task-related constraints, hence achieve specific tactical learning objectives (40) and provide a variety of options for adapting exercises and game formats. Scanning cues can be added to existing drills to enhance perceptual demands. The flexibility of the proposed game formats (see Table 1) enables coaches to adapt the content to suit their players’ developmental stage and training environment. For elite players who often possess a solid foundation of perception and motor skills, incorporating scanning task into SSGs appears to be an effective method of refinement. In contrast, grassroots players may first benefit from more foundational training in motor control and perception-action coupling before similar interventions produce comparable improvements.

This study aimed to initiate the establishment of evidence-based long-term training methods for scanning behavior in sports. Future research should investigate further age categories to provide a more comprehensive understanding of the development of scanning behavior across the career of female football players. While the game formats and exercises were well received by both players and coaches, the results obtained from the SSGs in the test sessions still need to be confirmed during 11v11 match play.

## 5 Conclusion

In conclusion, this study demonstrated that a five-week scanning-focused training intervention significantly improved scanning behavior in youth female football players, which was maintained in the three-week retention test. However, improvements were primarily observed in elite players, showing that the skill level affects the effectiveness of such a training intervention. Despite increased scanning behavior, no significant changes in successful subsequent actions were found. For coaches, this emphasizes the value and sustainability of integrating scanning-focused exercises within representative training environments even across a fairly short (five-week) intervention period.

## Data Availability

The datasets presented in this study can be found in online repositories. The names of the repository/repositories and accession number(s) can be found below: Zenodo: https://doi.org/10.5281/zenodo.15050638.

## Supplementary material

Supplementary Data Sheet 1 Playbook intervention group.

Supplementary Data Sheet 2 Playbook control group.

## References

[1] McGuckian TB Cole MH Jordet G Chalkley D Pepping GJ. Don't turn blind! the relationship between exploration before ball possession and on-ball performance in association football. Front Psychol. (2018) 9:2520. 10.3389/fpsyg.2018.02520 30618946 PMC6295565

[2] Jordet G Aksum KM Pedersen DN Walvekar A Trivedi A McCall A Scanning, contextual factors, and association with performance in English premier league footballers: an investigation across a season. Front Psychol. (2020) 11:553813. 10.3389/fpsyg.2020.553813 33123039 PMC7573254

[3] Aksum KM Pokolm M Bjorndal CT Rein R Memmert D Jordet G. Scanning activity in elite youth football players. J Sports Sci. (2021) 39(21):2401–10. 10.1080/02640414.2021.1935115 34078235

[4] Elderidge D Pulling C Robins M. Visual exploratory activity and resultant behavioural analysis of youth midfield soccer players. J Hum Sport Exerc. (2013) 8(3):S560–S77. 10.4100/jhse.2013.8.Proc3.02

[5] Jordet G Bloomfield J Heijmerikx J. The hidden foundation of field vision in English premier league soccer players. MIT sloan Sports Analytics Conference; Boston, MA (2013).

[6] Hintermann M Romann M Schmid J Taube W Fuchslocher J. The influence of scanning behaviour on performance during 4v4 small-sided games in youth female football. J Sports Sci. (2024) 42(21):1977–85. 10.1080/02640414.2024.2421662 39472553

[7] Pokolm M Kirchhain M Müller D Jordet G Memmert D. Head movement direction in football - a field study on visual scanning activity during the uefa-U17 and -U21 European championship 2019. J Sports Sci. (2023) 41(7):695–705. 10.1080/02640414.2023.2235160 37440444

[8] Caso S McGuckian TB van der Kamp J. No evidence that visual exploratory activity distinguishes the super elite from elite football players. Sci Med Footb. (2024) 9(2):172–80. 10.1080/24733938.2024.2325139 38451112

[9] Caso S Kamp J Morel P Savelsbergh G. The relationship between amount and timing of visual exploratory activity and performance of elite soccer players. Int J Sport Psychol. (2023) 54:287–304. 10.7352/IJSP.2023.54.287

[10] Phatak A Gruber M. Keep your head up—correlation between visual exploration frequency, passing percentage and turnover rate in elite football midfielders. Sports. (2019) 7(6):139. 10.3390/sports7060139 31174322 PMC6628054

[11] Pokolm M Rein R Muller D Nopp S Kirchhain M Aksum KM Modeling Players’ scanning activity in football. J Sport Exerc Psychol. (2022) 44(4):263–71. 10.1123/jsep.2020-0299 35468590

[12] Pedersen AV Aksdal IM Stalsberg R. Scaling demands of soccer according to anthropometric and physiological sex differences: a fairer comparison of men’s and women’s soccer. Front Psychol. (2019) 10:762. 10.3389/fpsyg.2019.00762 31024399 PMC6465549

[13] Legault I Faubert J. Gender comparison of perceptual-cognitive learning in young athletes. Sci Rep. (2024) 14(1):8635. 10.1038/s41598-024-59486-6 38622179 PMC11018768

[14] Yongtawee A Woo M-J. The influence of gender, sports type and training experience on cognitive functions in adolescent athletes. Exerc Sci. (2017) 26:159–67. 10.15857/ksep.2017.26.2.159

[15] UEFA. Uefa Annual Report 2022/23. (2024).

[16] Pfister G. Assessing the sociology of sport: on women and football. Int Rev Sociol Sport. (2015) 50:563–9. 10.1177/1012690214566646

[17] Feist J Datson N Runswick OR Harkness-Armstrong A Pocock C. Visual exploratory activity in elite women’s soccer: an analysis of the uefa women’s European championship 2022. Int J Sport Exerc Psychol. (2024) 23(2):281–303. 10.1080/1612197X.2023.2300386

[18] Pulling C Kearney P Eldridge D Dicks M. Football coaches’ perceptions of the Introduction, delivery and evaluation of visual exploratory activity. Psychol Sport Exerc. (2018) 39:81–9. 10.1016/j.psychsport.2018.08.001

[19] Eldridge D Pocock C Pulling C Kearney P Dicks M. Visual exploratory activity and practice design: perceptions of experienced coaches in professional football academies. Int J Sports Sci Coach. (2022) 18(2):370–81. 10.1177/17479541221122412

[20] Ometto L Vasconcellos FV Cunha FA Teoldo I Souza CRB Dutra MB How manipulating task constraints in small-sided and conditioned games shapes emergence of individual and collective tactical behaviours in football: a systematic review. Int J Sports Sci Coach. (2018) 13(6):1200–14. 10.1177/1747954118769183

[21] McGuckian T Askew G Greenwood D Chalkley D Cole M Pepping G-J. The Impact of Constraints on Visual Exploratory Behavior in Football. Abington, UK: Taylor & Francis (2017).

[22] Elderidge D Pulling C Robins M. The exploration of practice on youth soccer players visual exploratory activity. UK Coaching Applied Research Conference; 2014 19th February; Derby County Football Club, Pride Park Stadium; Derby, UK

[23] Dunton A O'Neill C Coughlan E. The impact of a training intervention with spatial occlusion goggles on controlling and passing a football. Sci Med Footb. (2019) 3:1–6. 10.1080/24733938.2019.1616106

[24] Dunton A O'Neill C Coughlan E. The impact of a spatial occlusion training intervention on pass accuracy across a continuum of representative experimental design in football. Sci Med Footb. (2020) 4:269–77. 10.1080/24733938.2020.1745263

[25] Pinder RA Davids K Renshaw I Araújo D. Representative learning design and functionality of research and practice in sport. J Sport Exerc Psychol. (2011) 33(1):146–55. 10.1123/jsep.33.1.146 21451175

[26] Gibson JJ. The Ecological Approach to Visual Perception. Boston, MA, US: Houghton, Mifflin and Company (1979). p. 346.

[27] Rico-González M Pino-Ortega J Praça GM Clemente FM. Practical applications for designing soccer’ training tasks from multivariate data analysis: a systematic review emphasizing tactical training. Percept Mot Skills. (2022) 129(3):892–931. 10.1177/00315125211073404 35084256

[28] Revelle W. Procedures for Psychological, Psychometric, and Personality Research. R Package Version 2.3.9 ed. Evanston, IL: Northwestern University (2023).

[29] Koo TK Li MY. A guideline of selecting and reporting intraclass correlation coefficients for reliability research. J Chiropr Med. (2016) 15(2):155–63. 10.1016/j.jcm.2016.02.012 27330520 PMC4913118

[30] Field A. Discovering Statistics Using Ibm Spss Statistics. Los Angels: Sage Publications Ltd (2013).

[31] Cohen J. Statistical Power Analysis for the Behavioral Sciences. 2nd ed. New York, NY: Routledge (1988).

[32] Farrow D Robertson S. Development of a skill acquisition periodisation framework for high-performance sport. Sports Med. (2017) 47(6):1043–54. 10.1007/s40279-016-0646-2 27873190

[33] Li L Smith DM. Neural efficiency in athletes: a systematic review. Front Behav Neurosci. (2021) 15:698555. 10.3389/fnbeh.2021.698555 34421553 PMC8374331

[34] Fernandes J Arida RM Gomez-Pinilla F. Physical exercise as an epigenetic modulator of brain plasticity and cognition. Neurosci Biobehav Rev. (2017) 80:443–56. 10.1016/j.neubiorev.2017.06.012 28666827 PMC5705447

[35] Chang Y. Reorganization and plastic changes of the human brain associated with skill learning and expertise. Front Hum Neurosci. (2014) 8:35. 10.3389/fnhum.2014.00035 24550812 PMC3912552

[36] Karim H Huppert T Erickson K Wollam M Sparto P Sejdic E Motor sequence learning-induced neural efficiency in functional brain connectivity. Behav Brain Res. (2017) 319:87–95. 10.1016/j.bbr.2016.11.021 27845228 PMC5183470

[37] Du Y He L Wang Y Liao D. The neural mechanism of long-term motor training affecting Athletes’ decision-making function: an activation likelihood estimation meta-analysis. Front Hum Neurosci. (2022) 16:854692. 10.3389/fnhum.2022.854692 35517985 PMC9062593

[38] Kantak SS Winstein CJ. Learning–performance distinction and memory processes for motor skills: a focused review and perspective. Behav Brain Res. (2012) 228(1):219–31. 10.1016/j.bbr.2011.11.028 22142953

[39] Wulf G McNevin N Shea C. The automaticity of Complex motor skill learning as a function of attentional focus. Q J Exp Psychol. (2001) 54:1143–54. 10.1080/713756012 11765737

[40] Davids K Araujo D Correia V Vilar L. How small-sided and conditioned games enhance acquisition of movement and decision-making skills. Exerc Sport Sci Rev. (2013) 41(3):154–61. 10.1097/JES.0b013e318292f3ec 23558693

