# Supplementary material for: “Heads Up Girls!” a training intervention to improve scanning behavior in youth female football
Source: Front Sports Act Living. 2025 Jun 27;7:1602099. doi: 10.3389/fspor.2025.1602099 (PMC12245709; doi:10.3389/fspor.2025.1602099)

Supplementary Material

1 Intervention group: game formats and exercises

1.1 Game format 1:

Teams: 4v4 with 3 neutral players (red)
Goals: None
Objective: The objective of the game is to maintain ball possession within the team (yellow and blue). When a neutral player outside of the field receives the ball, the other second neutral player holds up one of the two different colored cones. The player who receives the ball next must call out the color of the cone before her first touch of the ball. If she calls it too late, her team loses possession. If the ball leaves the pitch, the coach plays a new ball into play.

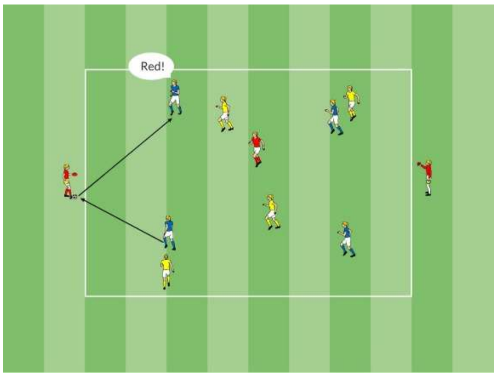

1.2 Game format 2:

Teams: 4v4 / 5v5
Goals: Two to three small goals per team
Objective: The teams each attack three small goals (basic form).

1. Variation: a neutral player (red) in the middle starts each new play for a quick transition after each goal
2. Variation: A line is marked 3-5m from the goal line, behind which a defender or a goalkeeper (neutral player in red) operates. This player spontaneously defends one of the goals as soon as the attacking team crosses the halfway line.
3. Variation: Change of game play after each goal.
4. Variation: Play with one-touch only

Basic form:

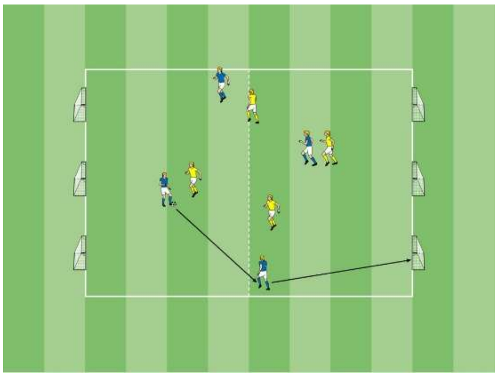

Variation 1:

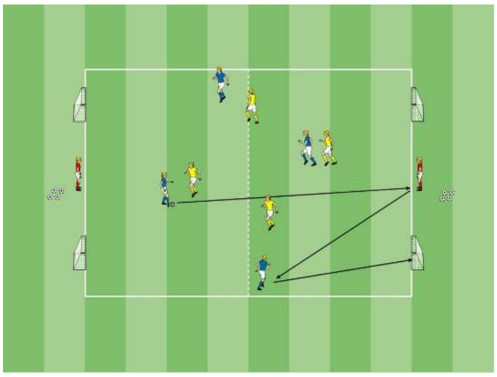

Variation 2:

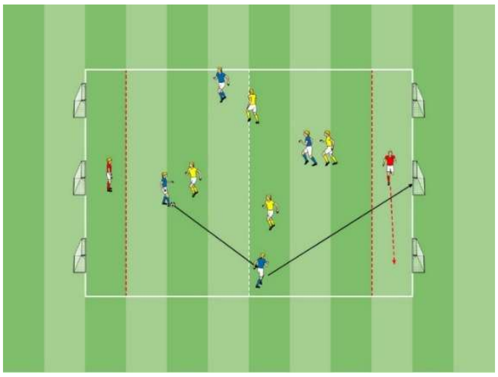

1.3 Game format 3:

Teams: 4v4 + 2 goalkeepers
Goals: Two regular-sized goals
Objective: The teams each attack one regular-sized goal. Once the ball crosses the halfway line, the attacking team can choose the direction of play freely. If ball possession changes (ball loss), the new team in possession must cross the halfway line again before attacking.

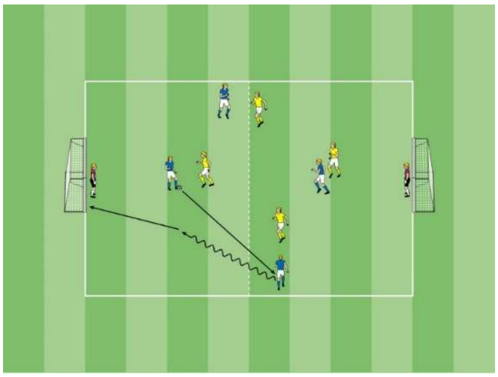

1.4 Game format 4:

Teams: 4v4 + 2 goalkeepers
Goals: Two regular-sized goals
Objective: The teams each attack one regular-sized goal. There are no throw-ins, corner kicks, free kicks, or other set-piece restarts. The game always restarts from the goalkeeper, who plays the ball to a teammate. The goalkeeper can only pass to teammates moving towards her (the goal line) and therefore facing away from the game play.

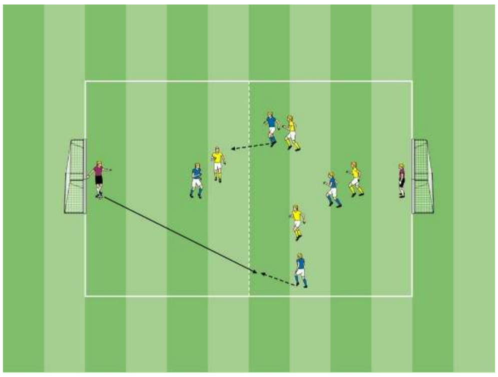

1.5 Exercise 1:

Teams: Groups of 6 players
Goals: None
Objective: A circle of 5 outside players, with the sixth player in the center. Two or three of the outside players have a ball. The player in the center demands a pass from the outside players and must pass it directly to another outside player (not in possession of a ball). The outside players can pass among themselves but must not disrupt the flow of the central player's movement (timing!).

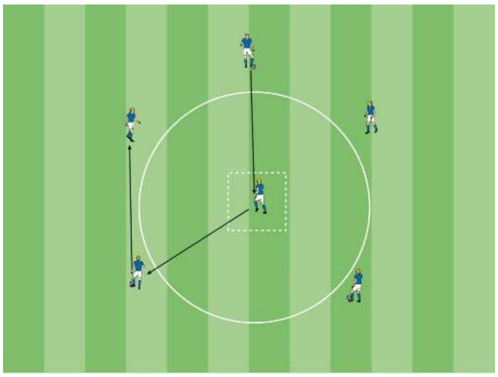

1.6 Exercise 2:

Teams: Groups of 3 players
Goals: None
Objective: Two players stand either side of a "cone wall" and pass the ball back and forth using 2-3 touches (focus: first touch). The ball is always received and played from opposite sides of the "cone wall". The third player occasionally plays a ball to one of the players, who must return it to them with just one touch while the passing drill continues.

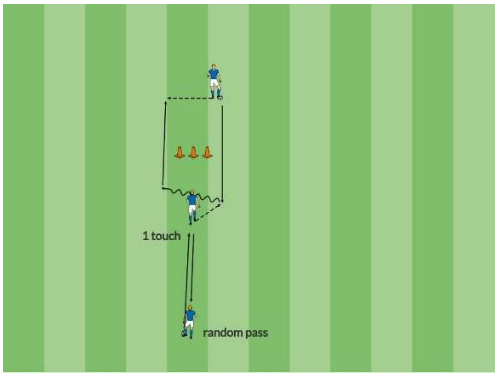

1.7 Exercise 3:

Teams: Groups of 4 players
Goals: None
Objective: The player in the center runs to the cone, which represents an opponent. The player behind her gives her a visual instruction by raising either her left or right arm. The player in the center adjusts her position accordingly to escape the opponent's influence (cone) and adopts a "3/4-position" (or open position). She then receives the ball from a third player and passes the ball on to the player who gave her the arm signal. The players rotate by following their pass. The fourth player now gives the arm signals to the new central player.

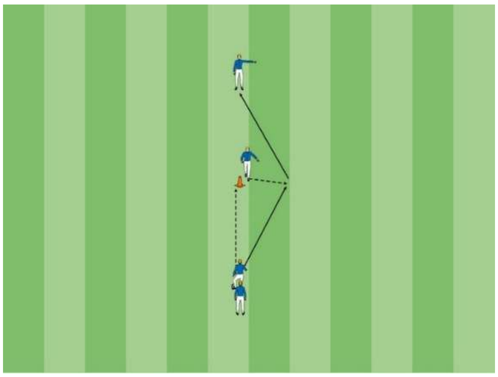

1.8 Exercise 4:

Teams: Three teams of 4 players
Goals: None
Objective: Each team has a ball and plays among themselves. The player passing the ball calls out the name of the player whom the receiver must pass to net.

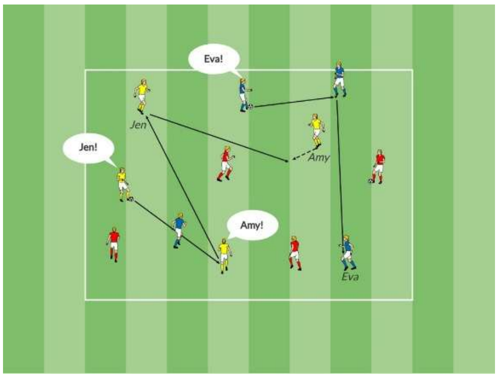

Supplement: Supplementary Data Sheet 1 — Playbook intervention group. [file Datasheet1.pdf]
