# Supplementary material for: “Heads Up Girls!” a training intervention to improve scanning behavior in youth female football
Source: Front Sports Act Living. 2025 Jun 27;7:1602099. doi: 10.3389/fspor.2025.1602099 (PMC12245709; doi:10.3389/fspor.2025.1602099)

Supplementary Material

1 Control group: game formats and exercises

1.1 Game format 1:

Teams: 2v2 with two passing options each
Goals: Two small goals per team
Objective: A 2v2 is played within the field. Each team has two passing stations positioned on the sideline in the offensive half. When they receive a pass, they enter the field (focus: first ball contact forward). The player who made the pass then rotates to the outside station.

1. Variation: Closer goal blocked for outside stations or only possible by direct shot
2. Variation: Multiple mini goals or regular-sized goals with goalkeeper on a bigger field

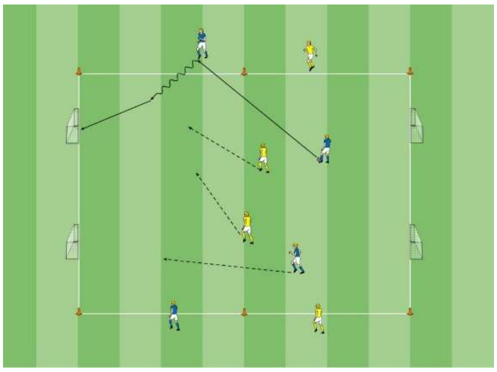

1.2 Game format 2:

Teams: 4v4
Goals: Three small goals per team / two regular-sized goals (with goalkeeper)
Objective: Normal game without special rules

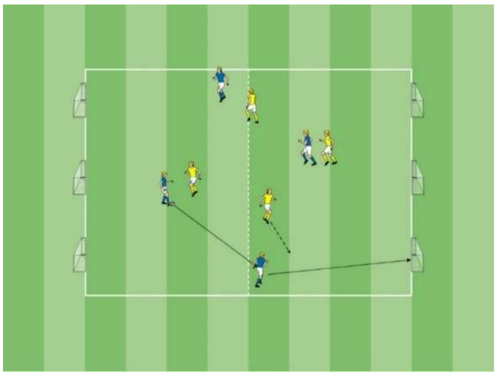

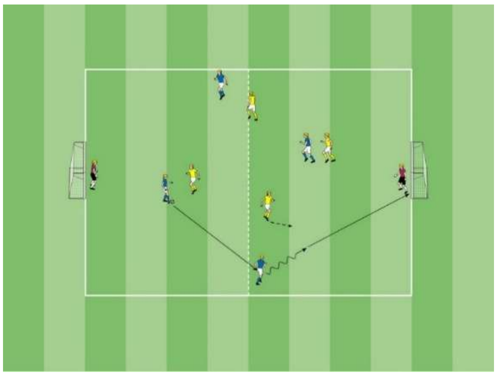

1.3 Exercise 1:

Teams: Groups of 3-4 players
Goals: None
Objective: 1) The players perform different tasks and receive passes (create different angles, depending on the practice setting). The following execution options: a) Ball reception in front and pass with the 2nd contact, b) Ball reception behind and pass with the 2nd contact, c) Ball reception to the inside and dribbling with a feint before the pass. 2) All the players are moving and process the ball while running.

1) 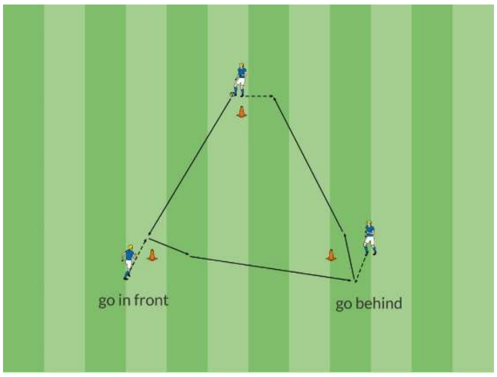

2) 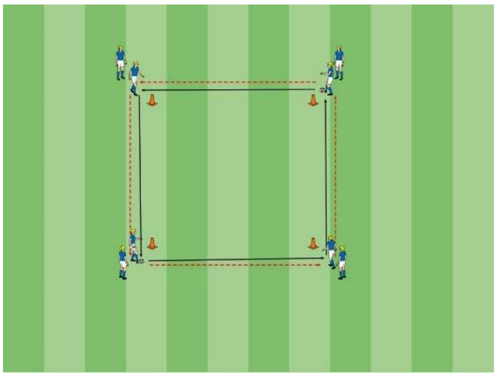

1.4 Exercise 2:

Teams: Two teams of 4 players
Goals: None; small zones marked with cones
Objective: Both teams have a ball and try to maintain ball possession within the team. A pass may only be played into one of the marked zones, where a teammate has to run in and takes the ball. The player must take the ball with the first contact a) 90° to the right, b) 90° to the left, c) forwards (180°) and leave the zone directly.

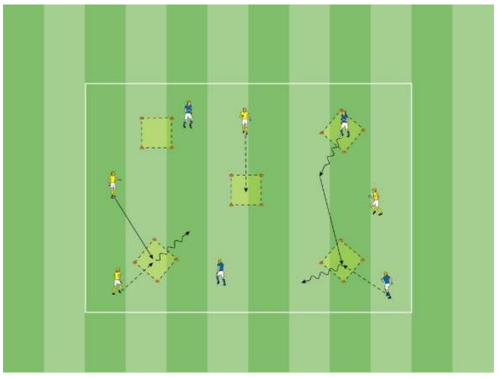

1.5 Exercise 3:

Teams: Groups of 4 players
Goals: None, two neutral players (red)
Objective: A 1v1 is played within the field. The game starts with a pass from a neutral player to the player in blue. This player then attempts to pass the ball to the opposite neutral player while playing 1v1 against the yellow player. She can either dribble past the opponent or attack the open space. Focus: Direction of the first touch.

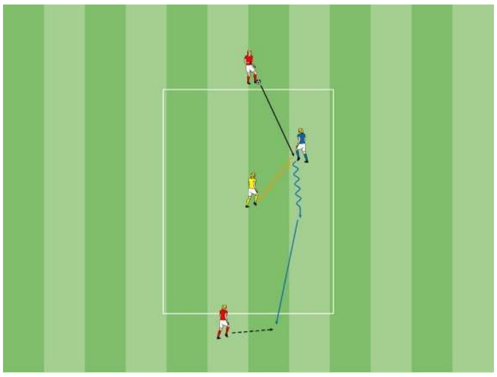

Supplement: Supplementary Data Sheet 2 — Playbook control group. [file Datasheet2.pdf]
